# Spontaneous Phage Resistance in Avian Pathogenic *Escherichia coli*

**DOI:** 10.3389/fmicb.2021.782757

**Published:** 2021-12-13

**Authors:** Patricia E. Sørensen, Sharmin Baig, Marc Stegger, Hanne Ingmer, An Garmyn, Patrick Butaye

**Affiliations:** ^1^Department of Pathobiology, Pharmacology and Zoological Medicine, Ghent University, Merelbeke, Belgium; ^2^Department of Biomedical Sciences, Ross University School of Veterinary Medicine, Basseterre, Saint Kitts and Nevis; ^3^Department of Bacteria, Parasites and Fungi, Statens Serum Institut, Copenhagen, Denmark; ^4^Department of Veterinary and Animal Sciences, University of Copenhagen, Frederiksberg, Denmark

**Keywords:** bacteriophage, phage resistance, phage-host interaction, *Eschericha coli*, phage therapy

## Abstract

Avian pathogenic *Escherichia coli* (APEC) is one of the most important bacterial pathogens affecting poultry worldwide. The emergence of multidrug-resistant pathogens has renewed the interest in the therapeutic use of bacteriophages (phages). However, a major concern for the successful implementation of phage therapy is the emergence of phage-resistant mutants. The understanding of the phage-host interactions, as well as underlying mechanisms of resistance, have shown to be essential for the development of a successful phage therapy. Here, we demonstrate that the strictly lytic *Escherichia* phage vB_EcoM-P10 rapidly selected for resistance in the APEC ST95 O1 strain AM621. Whole-genome sequence analysis of 109 spontaneous phage-resistant mutant strains revealed 41 mutants with single-nucleotide polymorphisms (SNPs) in their core genome. In 32 of these, a single SNP was detected while two SNPs were identified in a total of nine strains. In total, 34 unique SNPs were detected. In 42 strains, including 18 strains with SNP(s), gene losses spanning 17 different genes were detected. Affected by genetic changes were genes known to be involved in phage resistance (outer membrane protein A, lipopolysaccharide-, O- antigen-, or cell wall-related genes) as well as genes not previously linked to phage resistance, including two hypothetical genes. In several strains, we did not detect any genetic changes. Infecting phages were not able to overcome the phage resistance in host strains. However, interestingly the initial infection was shown to have a great fitness cost for several mutant strains, with up to ∼65% decrease in overall growth. In conclusion, this study provides valuable insights into the phage-host interaction and phage resistance in APEC. Although acquired resistance to phages is frequently observed in pathogenic *E. coli*, it may be associated with loss of fitness, which could be exploited in phage therapy.

## Introduction

Bacteriophages (phages) are viruses that specifically infect bacteria, and are estimated to be the most abundant organisms on Earth with more than 10^31^ entities (Güemes et al., 2016). Phages are unable to replicate independently of a susceptible bacterial host, and their host-range is determined by a combination of various factors, including specificity of host-binding phage proteins and bacterial phage-resistance mechanisms (Clokie et al., 2011; Ross et al., 2016). Virulent phages are strict parasites of their host and confer a selective pressure on their host population through host cell lysis (Buckling and Rainey, 2002). In response, bacteria can evolve resistance to phage infection through various mechanisms, such as spontaneous mutations, acquisition of restriction-modification (R-M) systems, and adaptive immunity via Clustered Regularly Interspaced Short Palindromic Repeats (CRISPR)-Cas system(s). These mechanisms can be used to target different steps of the phage life cycle, including phage attachment, adsorption, replication, and host cell lysis (Barrangou et al., 2007; Labrie et al., 2010). The different resistance mechanisms result in distinct resistance phenotypes. These can differ in whether the resistance is partial or complete, in the fitness cost associated with resistance, and in whether the mutation can be countered by a mutation in the infecting phage (Bohannan and Lenski, 2000; Weissman et al., 2018). Although various antiviral defense systems are found in bacteria, the emergence of phage resistance as well as phage-bacterium co-evolution are often driven by spontaneous mutations (Labrie et al., 2010; Koskella and Brockhurst, 2014), which may confer phage resistance by modifying phage-associated receptors on the bacterial surface. However, such changes have also been associated with reduced fitness relative to non-resistant strains (Azam and Tanji, 2019). Phage-resistant bacteria may become less virulent as in case when mutations occur in their lipopolysaccharides (LPS), or may experience impaired growth in case of mutations in genes involved in essential cell functions (Burmeister and Turner, 2020). Additionally, maintenance of defense systems such as R-M enzymes and CRISPR-Cas, also has its own costs associated with enzyme production and expression (Vasu and Nagaraja, 2013; Vale et al., 2015; Bradde et al., 2019).

Avian pathogenic *Escherichia coli* (APEC) is one of the most important bacterial pathogens affecting poultry. These pathogens cause a large range of extra-intestinal infections, which collectively are referred to as colibacillosis. These infections can result in high morbidity and mortality, and hereby, significant economic loses to the poultry industry worldwide (Dho-Moulin and Fairbrother, 1999; Zhao et al., 2005; Lutful Kabir, 2010; Nolan et al., 2013). Here, the APEC with O-serogroups O1, O2, and O78 constitute more than 80% of the infection cases (Kathayat et al., 2018). As current antimicrobials become increasingly inadequate to treat bacterial infections and a global focus to reduce conventional antimicrobial usage in general, alternative treatment strategies, such as the therapeutic use of phages (phage therapy), are urgently needed (Centers for Disease Control and Prevention [CDC], 2013; World Health Organization [WHO], 2014; Lin et al., 2017). However, being able to understand phage-host interactions as well as the underlying mechanisms of resistance is essential for successful phage therapy application (Oechslin, 2018). Here, we investigate the phage-host interactions and resistance through isolation and characterization of spontaneous phage-resistant mutants of APEC.

## Materials and Methods

### Bacterial Strains and Growth Conditions

The avian pathogenic *E. coli* (APEC) ST95 O1:H7 strain, AM621, is part of the in-house collection that was isolated from clinical material suspected of APEC infection from Belgium collected during 2013–2014 by Animal Health Care Flanders (Torhout, Belgium). The *E. coli* K-12 derived laboratory strain K514 (Colson et al., 1965) was included as a phage-susceptible control and host strain. Bacterial strains were grown in Luria Bertani (LB) broth or on LB agar supplemented with 1.5% bacteriological agar no. 1 (*w/v*) (Oxoid, Thermo Fisher Scientific, United States) overnight (16–18 h) at 37°C unless stated otherwise. Broth cultures were incubated with shaking (120 rpm). Strains were stored at −80°C in LB broth supplemented with 15% glycerol.

### Bacteriophage Isolation and Propagation

The strictly virulent *Escherichia* phage vB_EcoM-P10 (SRA accession no. SRX8360061) used in this study is a part of the in-house phage collection. The phage was isolated from poultry feces and processed as previously described (Sørensen et al., 2020). Phage lysates were stored at 4°C, at titers ranging from ∼1.2 × 10^8^ to 1.4 × 10^9^ plaque forming units (PFU)/ml. *Escherichia* phage vB_EcoM-P10 was classified (according to the International Committee on Taxonomy of Viruses (ICTV) taxonomy) as a tailed *Myoviridae* phage belonging to the *Tevenvirinae* subfamily and *Tequatrovirus* genus.

### Isolation of Phage-Resistant Mutant Strains

Phage-resistant APEC strains were obtained using the agar plate (AP) (Reinheimer et al., 1995) and the secondary culture (SC) technique (Carminati et al., 1993) with minor modifications (Supplementary Figure 1). Briefly, overnight culture of wildtype (WT) strain AM621 was inoculated in LB broth supplemented with CaCl_2_ (final concentration of 10 mM) and then infected with suspension of virulent phage vB_EcoM-P10, at a multiplicity of infection (MOI) of 0.1, 1, 10, and 100. For the AP technique, suspensions were streaked directly onto LB agar plates supplemented with CaCl_2_ (final concentration of 10 mM) and incubated for 48 h at 37°C. After incubation of 24 and 48 h, individual colonies were selected from each MOI suspension and cultured in LB broth. Isolates were purified by three consecutive streakings on LB agar and recovered as presumptive phage-resistant mutants. Remaining MOI cultures that were not streaked on agar plates were subjected to the SC technique. Cultures were incubated at 37°C with shaking (120 rpm) for ∼5 h. Cultures exhibiting complete or partial lysis and subsequent (secondary) growth after an additional incubation of 24 h were selected and streaked on LB agar plates. Remaining “SC-T24” solutions were stored at 4°C until required. Presumptive phage-resistant mutants were recovered as described for the AP technique and stored at 4°C until required. An experiment with phage-susceptible *E. coli* laboratory strain K514 was performed in parallel as control. The AP/SC experiments were repeated six times.

Presumptive phage-resistant mutants were infected with phage vB_EcoM-P10 using the fitness test experimental set-up (described below). Mutants that displayed normal bacterial growth or increased growth compared to the phage-sensitive AM621 WT strain were defined as true phage-resistant mutants and stored at -80°C in LB broth supplemented with 15% glycerol (*v/v*). Efficiency of the phage-resistant mutant recovery was calculated according to the formula presented by Capra et al. (2011): (number of true phage-resistant mutants / number of presumptive phage-resistant mutants) * 100.

### Isolation and Enumeration of Potential Phage Mutants

To isolate potential phage mutants, the SC-T24 solutions were centrifuged and filtered using a 0.2 μm filter (Whatman, GE Healthcare, Germany). The filtrated SC-T24-phage suspensions were enumerated and tested for lytic activity on the host bacteria, *E. coli* K-12 derived laboratory strain K514, using the double-layer agar (DLA) technique (Kropinski et al., 2009). Briefly, phage suspensions were serial diluted and spotted on an overlay of the host bacteria on LB agar supplemented with 0.7% agar and 0.5 mM CaCl_2_. A clear zone in the plate, a plaque, resulting from the lysis of host bacterial cells, indicated the presence of virulent phage. Phage lysates were stored at 4°C until required.

### Bacterial Fitness

Bacterial reduction experiments were performed as described previously (Xie et al., 2018; Storms et al., 2020), with minor modifications. Bacterial overnight cultures were used, and the cell concentration was adjusted to ∼10^8^ colony forming units (CFU)/ml for every experiment. Bacterial suspensions were inoculated with phage, yielding MOIs of 0.1, 1, 10, and 100. All bacterial reduction curves were generated using 96-well plates with working volumes of 200 μl. The experiment was carried out in duplicates and repeated three times. Two wells of phage-free bacterial cultures and two wells of bacteria-free phage culture were included on every plate as control experiments in addition to one media blank for reference. Optical density (OD) for the wavelength of 600 nm was measured with the Thermo Fisher Scientific Multiskan GO Microplate Spectrophotometer and the data were recorded using the SkanIt Software, v6.0.2.3. OD600 measurements were taken immediately after inoculation and then at 30 min intervals afterward for 22 h. The protocol parameters included incubation temperature of 37°C and continuous shaking with medium speed. Reduction curves were obtained by plotting OD600 values after baseline adjustments against time. For each reduction curve, area under the curve (AUC) was calculated using GraphPad Prism v9.1.0.221 with default settings. AUC was calculated as average of four replicates. Strains were defined as truly resistant when % of decrease in AUC in the presence of phage was minimum 20% less relative to the WT strain. Fitness cost associated with acquired mutations in true resistant strains was defined as decrease in AUC compared to WT strain in the absence of phage.

### Genomic DNA Extraction and Sequencing

Genomic DNA was extracted from true phage-resistant bacterial strains using Qiagen’s DNeasy Blood and Tissue Kit (Qiagen, Hilden, Germany), with subsequent library construction using the Nextera XT Kit (Illumina, Little Chesterford, United Kingdom) using a 300-cycle kit on the Illumina NextSeq 550 platform according to the manufacturer’s instructions.

Phage DNA was extracted and purified using Phage DNA Isolation Kit (Norgen Biotek Corp., Canada), as indicated by the instructions provided by the manufacturer. The DNA yield was quantified using the QuantiFluor dsDNA System (Promega) and Quantus Fluorometer. The DNA purity (OD 260/280 ratio of ∼1.7–1.8) was measured using NanoDrop (Isogen Life Science). Libraries were constructed using the Nextera XT Kit (Illumina, Little Chesterford, United Kingdom) using a 300-cycle kit on the Illumina NextSeq platform according to the manufacturer’s instructions.

### Bacterial Genome Analysis

The open-source bifrost software,^1^ v1.1.0, was used for quality control of the WGS data. The raw reads were *de novo* assembled using SPAdes v3.11.1 (Bankevich et al., 2012), and contigs with less than 200 bp were excluded. APEC serotype was predicted for each of the strains using SerotypeFinder, v2.0 (Joensen et al., 2015). Genomes were annotated using Prokka, v1.12 (Seemann, 2014), and pan genome analysis was carried out with Roary, v.3.12.0 (Page et al., 2015), with minimum 90% similarity on protein level. Gene presence was subsequent confirmed using Mykrobe predictor, v0.5.6 (Bradley et al., 2015). Genes classified as present were further filtered for coverage (c > 70) and depth (d > 3). When inconsistencies were observed, manual BLAST searches were performed. Cases where a gene was detected in a mutant strain but not in the WT strain were excluded from further analysis, as this was assumed to be sequencing error or contamination (a false-positive).

PlasmidFinder 2.1 with default settings was used to screen assembled genomes for plasmids in the *Enterobacteriaceae* database. Plasmid replicons with less than 90% identity and 60% coverage were excluded. ABRicate v1.0.1^2^ with default options was used to screen assembled genomes for antimicrobial resistance genes with ResFinder database (Zankari et al., 2012), NCBI Bacterial Antimicrobial Resistance Reference Gene Database (Feldgarden et al., 2019), and the Comprehensive Antibiotic Resistance Database (CARD) (Alcock et al., 2019). Virulence genes were identified using ABRicate with sequences from the Ecoli_VF database.

Clustered Regularly Interspaced Palindromic Repeats (CRISPR) systems were identified using the Geneious Prime v2020.1.1 Crispr Recognition Tool Wrapper (CRT) tool v1.1. and CRISPRCasFinder^3^ (Couvin et al., 2018) with default settings. A quality score was automatically given to CRISPR arrays consisting of repeats and spacer sequences in the form of “evidence level,” rated 1–4, where 1 includes small CRISPRs (with three or less spaces) and 2–4 are classified based on repeat and spacer similarity. BLAST analysis was performed to determine if identified CRISPR spacer sequences matched the invading *Escherichia* phage vB_EcoM-P10 genome.

### Bacterial Core Genome Single Nucleotide Polymorphism Analysis

To assess the relationship between strains, a single nucleotide polymorphism (SNP)-based phylogeny was obtained using SNPs identified by the Northern Arizona SNP Pipeline (NASP), v1.2.0 (Sahl et al., 2016), with the Burrows-Wheeler Aligner (BWA) algorithm, v0.7.17-r1188 (Li and Durbin, 2009). Illumina reads from all individual strains were aligned against the AM621 WT scaffold genome obtained as described with a cutoff of all contigs < 500 bp above after removal of duplicated regions using NUCmer, v3.1 (Marçais et al., 2018). Positions with less than 10-fold coverage and less than 90% unambiguous variant calls were excluded across the collection. The chromosome from the well-characterized ST95 *E. coli* isolate UTI89 (GenBank accession number NC_007946) was used to infer functionality of all the identified SNP differences.

### Phage Genome Analysis

Phage genome analysis, including quality control validation, *de novo* assembly, annotation, and pan genome analysis, was performed as described above for the bacterial genomes. Core genome SNP analysis was performed as described for the bacterial genome using the chromosome from the highly similar, well-characterized *Escherichia* phage vB_EcoM_G29 (GenBank accession number MK327940) as reference.

## Results

### Isolation of Phage-Resistant Mutants

A total of 264 presumptive phage-resistant variants were obtained from the AP and SC methods using the strictly virulent *Myoviridae* phage vB_EcoM-P10. Only 109 isolates (∼41%) were considered true phage-resistant derivatives based on increase in bacterial growth [area under the curve (AUC)] relative to the WT strain in the presence of phage. In this study, the SC method generated more mutants than the AP method (Table 1).

**TABLE 1:** Phage-resistant mutants isolated using secondary culture (SC) or agar plate (AP) methods.

	No. of presumptive phage resistant mutants	No. of true phage-resistant mutants	Isolation efficiency
AP	132	33	25%
SC	132	76	58%

For the AP method, the highest number of true resistant mutants were isolated from MOI 100 suspensions (∼42%) and the lowest from MOI 0.1 (0%). For the SC method, the highest number of true resistant mutants were isolated from MOI 1 suspensions (∼30%) and the lowest from MOI 100 (∼21%). Similar numbers of true resistant mutants were isolated after 24 and 48 h of incubation (Supplementary Figure 2).

### Bacterial Fitness

**FIGURE 1:**
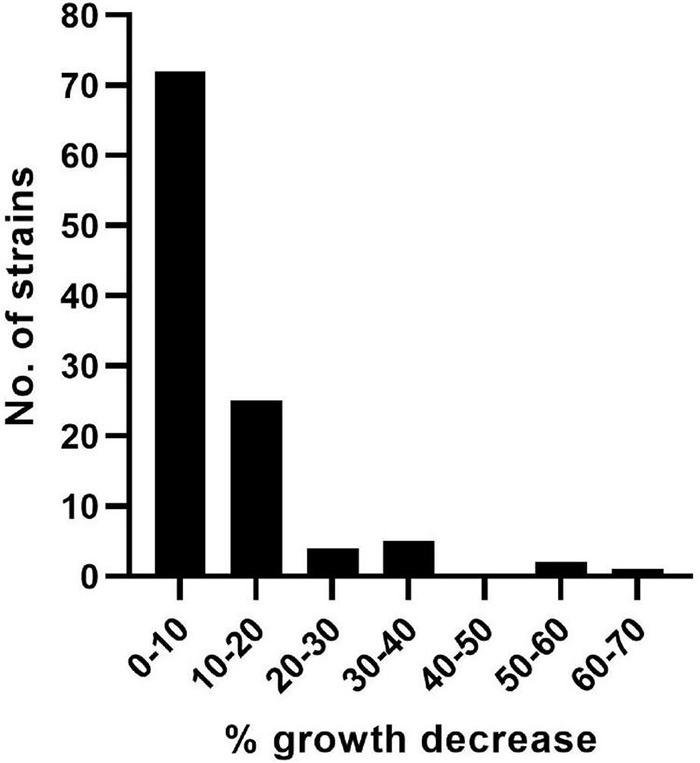
Decrease in growth of the phage-resistant APEC strains. The fitness cost associated with acquired genetic changes in phage-resistant strains was determined as percentage decrease in overall bacterial growth (area under the curve).

The fitness cost associated with acquired mutation(s) in phage-resistant strains was determined as decrease in overall bacterial growth (AUC) relative to the WT stain in the absence of phage (Supplementary Table 1). The greatest fitness cost was detected for mutant strain SC48_10_8 (65% growth reduction), followed by AP48_1_24 (59%) and SC24_01_5 (57%). A fitness cost of 31.6–37.5% was observed for five mutants. A fitness cost of 22.0–28.7% was observed for four mutants. A fitness cost of 10.4–18.8% was observed for 24 mutants. A fitness cost of 5.2–9.9% was observed for 33 mutants, and low or no fitness cost (< 5%) was observed for 39 of the mutant strains (Figure 1).

### Bacterial Genome Analysis

WGS of the bacterial genomes yielded a total of 1,934,298– 6,753,240 paired-end reads for each isolate with an average coverage of 51–177-fold. *De novo* assembly resulted in 192–353 contigs and an N50 value from between 51,335 and 189,445 bp.

**FIGURE 2:**
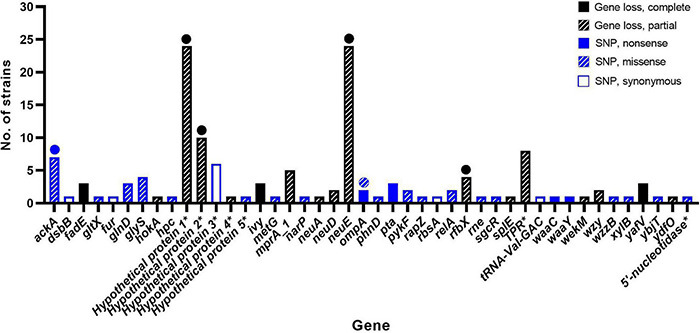
Genes affected by genetic changes in phage-resistant APEC strains. In total, 44 different genes were affected by genetic change(s). Genetic changes included complete gene loss, partial gene loss, or point mutations (nonsense, missense, or synonymous). Full circle = complete gene loss in few mutants or nonsense mutation in one mutant. Striped circle = missense mutation in one mutant. * = protein name is shown as gene name is unknown.

The bacterial strains were subjected to WGS analysis. All 109 resistant strains showed similar genetic characteristics as the AM621 WT, including a genome size between ∼5.27 and ∼5.40 Mbp and G+C content between 50.2 and 50.6%. Gene absence/presence analysis identified a total of 17 different accessory genes (after exclusion of false positives), that were lost (partial or complete) in one or more of mutant strains (Figure 2 and Table 2). A full overview of the genes lost in phage-resistant mutants is shown in Supplementary Table 2.

**TABLE 2:** Summary of genetic changes and affected genes in phage-resistant *E. coli* strains.

Change	Affected gene	Annotation	Function	No. of strains	Reference(s)
Gene loss	*neuE*	Polysialic acid biosynthesis protein, NeuE	*E. coli* K1 sialic acid capsule synthesis	24	Steenbergen and Vimr, 2008
Gene loss	*group_67*	Hypothetical protein	Unknown	24	–
Gene loss	*group_237*	Hypothetical protein	Unknown	10	–
Gene loss	*group_310*	Tetratricopeptide repeat (TPR) protein	Mediation of protein-protein interactions	8	Cerveny et al., 2013
SNP	*ackA*	Acetate kinase	Phosphorylation of acetate to acetyl phosphate	7	Schütze et al., 2020
SNP	SNP1*	Hypothetical protein	Unknown	6	–
Gene loss	*mprA_1*	MarR family transcriptional regulator	Regulation of numerous cellular processes	5	Deochand and Grove, 2017
Gene loss	*rfbX*	O-antigen transporter	Transport of O-polysaccharide molecules	4	Cowley et al., 2018
SNP	*glyS*	Glycyl-tRNA synthetase beta chain	tRNA recognition	4	Nagel et al., 1984
Gene loss	*fadE*	Acyl-CoA dehydrogenase	Dehydrogenation of acyl-coenzymes A	3	Campbell and Cronan, 2002
Gene loss	*yafV*	YafV (2-oxoglutaramate amidase)	Metabolite repair enzyme	3	Peracchi et al., 2017
Gene loss	*ivy*	Vertebrate lysosome inhibitor	Protection against lysozyme-mediated cell wall hydrolysis	3	Deckers et al., 2008
SNP	*glnD*	Protein-PII uridylyltransferase	Nitrogen regulation	3	Zhang et al., 2010
SNP	*pta*	Phosphate acetyltransferase	Acetate metabolism	3	Schütze et al., 2020
Gene loss	*epsM_1*	Acetyltransferase/NeuD protein	*E. coli* K1 sialic acid capsule synthesis	2	Daines et al., 2000
Gene loss	*wzy*	O1 family O-antigen polymerase	Synthesis of the LPS B-band O antigen	2	Wright et al., 2019
SNP	*ompA*	Outer membrane protein A (OmpA)	Key *E. coli* virulence factor	2	Smith et al., 2007; Bertozzi Silva et al., 2016
SNP	*relA*	GTP pyrophosphokinase	Synthesis of ppGpp from GTP	2	Korch et al., 2003
SNP	*pykF*	Pyruvate kinase	Regulation of the glycolytic pathway	2	Valentini et al., 2000
Gene loss	*wekM*	Glycosyltransferase family 4	Peptidoglycan biosynthesis	1	Wang et al., 2015; Cowley et al., 2018
Gene loss	*group_271*	Hypothetical protein	Unknown	1	–
Gene loss	*hokA*	HokA	Toxin of a type I toxin-antitoxin (TA) system	1	Pedersen and Gerdes, 1999
Gene loss	*ydfO*	DUF1398 family protein, YdfO	Unknown	1	Frederix et al., 2014
Gene loss	*neuA*	Acylneuraminate cytidylyltransferase	*E. coli* K1 sialic acid capsule synthesis	1	Daines et al., 2000
Gene loss	*splE*	Serine protease SplE	Involved in various biological processes	1	Stach et al., 2018
SNP	*dsbB*	Periplasmic thiol:disulfide oxidoreductase DsbB	Electron transfer catalyst	1	Grauschopf et al., 2003
SNP	*wzzB*	O-antigen chain length determinant protein WzzB	Lipopolysaccharide (LPS) biosynthesis	1	Stenberg et al., 2005
SNP	*metG*	Methionyl-tRNA synthetase	Protein biosynthesis	1	Deniziak and Barciszewski, 2001
SNP	SNP11*	tRNA-Val-GAC	Transfer of amino acids to the ribosome	1	Shepherd and Ibba, 2015
SNP	*narP*	Nitrate/nitrite response regulator protein NarP	Gene expression regulation	1	Noriega et al., 2010
SNP	*gltX*	Glutamyl-tRNA synthetase	Protein biosynthesis	1	Breton et al., 1986
SNP	*hcp*	T6SS component Hcp	Bacterial interaction with host cells	1	Navarro-Garcia et al., 2019
SNP	*rapZ*	RNase adapter protein RapZ	Cell envelope precursor sensing and signaling	1	Khan et al., 2020; Mutalik et al., 2020; Zhou et al., 2021
SNP	*rbsA*	Monosaccharide-transporting ATPase	Transfer of solutes across membranes	1	Keseler et al., 2017
SNP	*xylB*	Xylulose kinase	Phosphorylation of D-xylulose to D-xylulose 5-phosphate	1	Di Luccio et al., 2007
SNP	*waaC*	Lipopolysaccharide core heptosyltransferase I	Lipopolysaccharide (LPS) biosynthesis	1	Wang et al., 2015
SNP	*waaY*	Lipopolysaccharide core heptose (II) kinase RfaY	Lipopolysaccharide (LPS) biosynthesis	1	Wang et al., 2015
SNP	*fur*	Ferric uptake regulation protein FUR	Transcriptional regulation of iron metabolism	1	Seo et al., 2014
SNP	SNP31*	5’-nucleotidase	Hydrolysis of the phosphate group of 5′-nucleotides	1	Terakawa et al., 2016
SNP	*phnD*	Phosphonate ABC transporter substrate-binding protein PhnD	Phosphonate uptake and utilization pathway	1	Alicea et al., 2011
SNP	*ybjT*	Uncharacterized protein YbjT	*E. coli* LPS biosynthesis and other core cell envelope components	1	Al Mamun et al., 2012
SNP	*rne*	Ribonuclease E	RNA processing and mRNA degradation	1	Khemici et al., 2008
SNP	*sgcR*	sgc region transcriptional regulator	Transcriptional regulation	1	Magnet et al., 1999
SNP	SNP37*	Hypothetical protein	Unknown	1	–

* = gene not determined. Specified genetic change is included instead.

None of the mutant strains lost any plasmid replicons compared to the WT. The six plasmid replicons detected included Col(MG828), IncFIA, IncFIB(AP001918), IncFIC(FII), IncI1-I(Alpha), and IncX1. All but one mutant strain encoded the same resistance genes as the WT strain. Only one mutant strain, AP24_100_8, had lost *qnrS1*, a quinolone resistance gene. A total of 226 different virulence genes were all identified in both the WT strain and all the mutants.

Two different type I-F CRISPR systems (evidence level 4) were detected in the AM621 WT strain. The first system comprised seven repeat units of 20 bp and six CRISPR spacers, including five spacers of 40 bp and one spacer of 41 bp. The second system comprised six repeat units of 28 bp and five spacers of 32 bp. Moreover, two additional small CRISPR-like structures (evidence level 1); one with only two CRISPR repeats (44 bp) and one spacer (52 bp) and another with only two repeats (36 bp) and one spacer (59 bp) were separately identified in the genome. The same two CRISPR systems and two small CRISPR-like elements were found in all 109 mutant strains. Additionally, between one and eight evidence level one CRISPR-like structures, which were not in the WT strain, were detected in 102 of the mutants (Supplementary Table 3). Only three mutant strains, AP24_10_14, AP48_1_24, and SC24_01_5, had acquired a CRISPR-like element spacer of 53 bp that matched the invading phage genome.

### Bacterial Core Genome Single Nucleotide Polymorphism Analysis

**FIGURE 3:**
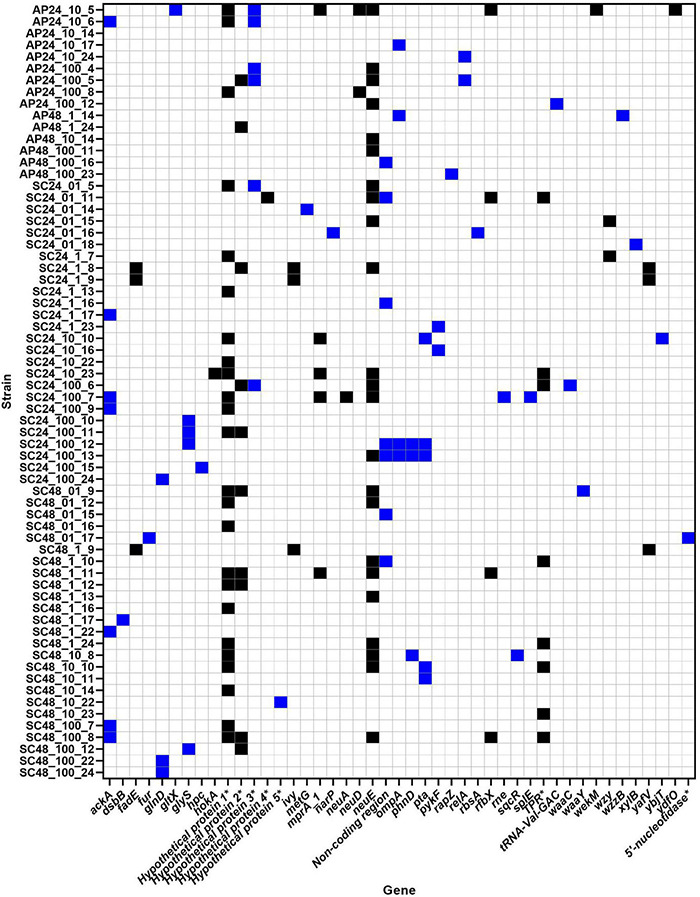
Phage-resistant strains and their genetic changes. In total, 44 different genes were affected by genetic change(s). Blue = SNP mutation. Black = partial and/or complete gene loss. * = protein name is shown as gene name is unknown.

SNP analysis identified between 0 and 2 SNP difference(s) in the core genome between AM621 and the mutants. Of the 109 mutants, 66 showed no SNP differences, 33 mutants showed one SNP difference and 10 mutants showed two SNP differences (Figure 3). A summary of SNPs identified in the mutants is shown in Table 2 and Figure 2. The specific amino acid change information is shown in Supplementary Table 2. A total of 37 unique SNPs were identified, five of which resulted in a nonsense mutation, 21 in a missense mutation, six in a synonymous mutation, and five of which were found in non-coding regions when analyzed against the annotation of the UTI89 genome. Nonsense mutations were found in five different genes, including acetate kinase (*ackA*), outer membrane protein A (*ompA*), phosphate acetyltransferase (*pta*), LPS core heptosyltransferase I (*waaC*), and LPS core heptose (II) kinase (*waaY*) (Figure 2). Missense mutations were found in 19 different genes (Supplementary Table 2).

### Impact of Selection Methods on Mutations

**FIGURE 4:**
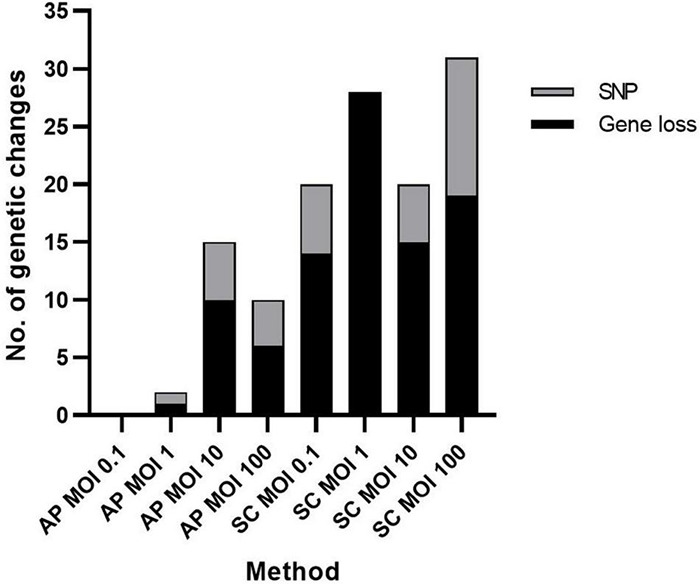
Type of bacterial genetic change detected for each method and multiplicity of infection (MOI). The number and type of genetic changes including SNP (gray) and partial or complete gene loss (black) organized based on method used. AP, agar plate; SC, secondary culture, at the four different MOIs: 0.1, 1, 10, and 100.

Number and type of genetic changes (gene loss or SNP) in the phage-resistant mutant strains was compared in relation to selection method (AP or SC), including the four different MOIs, 0.1, 1, 10, and 100 (Figure 4). The SC method produced the highest number of genetic changes. No genetic changes were detected in resistant strains generated using the AP-MOI-0.1 selection method. For all other selection methods, gene loss was the dominant type of genetic change, with the only exception of AP-MOI-1 where both gene loss and SNP were detected once.

### Phage Genome Analysis

To investigate if the 24 co-cultured SC-24 phages had evolved to overcome phage resistance mechanisms in the mutant strains, these phages, as well as the WT *Escherichia* phage vB_EcoM-P10, were subjected to WGS. The WGS of the phage genomes yielded a total of 942,276–2,338,994 paired-end reads for each isolate with an average coverage of 794–1,998-fold. *De novo* assembly resulted in 18–206 contigs and an N50 value ranging from 167,139 to 167,243 bp. Pan-genome analysis of the 25 *E. coli*-infecting phages (coliphages) included 271 genes. All genes were detected in all potential mutant phages using BLAST. SNP analysis identified no SNP differences in the core genome between WT *Escherichia* phage vB_EcoM-P10 and the potential phage mutants.

## Discussion

In this study, we selected and characterized phage-resistant mutant strains of O1 APEC strain AM621. Using a combined approach of the SC and AP method resulted in an overall mutant isolation efficiency of ∼41%. Previous studies using this approach found an isolation efficiency of true resistant *Lactobacillus paracasei* isolates of 56% (Sunthornthummas et al., 2019) and an average isolation efficiency of 36.5% (ranging between 29.5 and 50%) of true resistant *Lactobacillus delbrueckii* isolates (Guglielmotti et al., 2006). We found an SC method isolation efficiency of 57.6%, while the AP method efficiency was much lower (25.0%). The higher efficiency of the SC method has been reported before though with similar, smaller or larger differences (Guglielmotti et al., 2006; Binetti et al., 2007; Sunthornthummas et al., 2019). The lower AP efficiency (especially at low MOIs) could be explained by a low selection pressure for phage resistance. When comparing the specific isolation percentages, one must take into consideration the differences in how “true resistance” was defined as well as the differences of the bacterial WT strains used. In our study, the resistant mutants were quantitatively defined (increased AUC relative to the WT strain in the presence of phage) whereas previous studies used a qualitative approach (visual comparison of turbidity between phage-host co-cultures and control culture) to define true resistance. As opposed to the qualitative approach, defining true resistant mutants based on AUC provide high-throughput assessment based on fixed cut-off values, which can easily be compared, and do not depend on experience and/or subjectivity of the observer. However, one must be aware of the potential pitfalls related to the AUC as selection criterion. If the AUC increase percentage cut-off value is too high, true resistant mutants may wrongfully be excluded. If the cut-off value is too low, this approach could select both resistant mutants and non-resistant strains.

Bacteria have been shown to evolve resistance to phage infection through mechanisms of adsorption inhibition, including loss or modification of phage receptors (Bohannan and Lenski, 2000; Labrie et al., 2010; Rostøl and Marraffini, 2019). There is a great diversity reported in coliphage receptors, which include bacterial outer membrane proteins (OMPs), porins, capsule and LPS (Bertozzi Silva et al., 2016; Hantke, 2020; Mutalik et al., 2020). OMPs participate in outer membrane functionality, including diffusion and transport mechanisms, cell shape as well as virulence (Wang, 2002). Also, the OmpA protein has been shown to be a key virulence factor of pathogenic *E. coli* playing a role in conjugation, adhesion, immune system evasion, resistance to environmental stress (Smith et al., 2007). Therefore, mutation in such gene, while conferring resistance, may decrease bacterial adhesion and immune system evasion, and hereby, the overall strain virulence *in vivo*/*in situ*. In addition, phage resistance may also have a fitness cost (Burmeister and Turner, 2020). In this study, we observed up to 65% decrease in *in vitro* fitness (bacterial growth) in mutant strains that had acquired resistance through genetic mutations and/or gene loss. However, such fitness cost may vary in *in vivo*/*in situ* environments, as the magnitude has been shown to depend on the genetic basis of the resistance as well as on the environmental context (Mangalea and Duerkop, 2020).

Recently, Maffei et al. (2021) investigated the coliphage-host interaction and identified phage receptors. In accordance with previous findings, *Myoviridae* coliphages belonging to the *Tequatrovirus* genus were found to use the OMP, Tsx (T6-like phages), FadL (T2-like phages), OmpA, OmpC (T4-like phages), or OmpF as primary receptor. A recent study similarly identified the OmpA protein as a *Myoviridae* coliphage receptor and reported that all phage-resistant strains had acquired mutations in just two pathways, the LPS biosynthesis and the OmpA expression (Salazar et al., 2021). LPS are known to play an essential role in the OMP folding and placement in the cell wall (Bulieris et al., 2003). Accordingly, loss or changes in the structure of LPS could prevent OmpA from being properly positioned in the outer membrane, and thereby, making the phage receptor unavailable. In our study, we detected SNPs in the *ompA* gene, encoding the OmpA protein, suggesting this could act as receptor for phage vB_EcoM-P10. However, further studies are needed to confirm if OmpA is the primary receptor as well as determine the indirect effects on infection due to LPS changes.

While for some phages the absence of the primary receptor results in complete absence of infection, other phages, including those utilizing several receptors, are still able to infect (Islam et al., 2019; Chen et al., 2020; Maffei et al., 2021). The specificity for the second receptor depends on the short tail fibers of which two variants have been described to date (Maffei et al., 2021). The first variant (encoded by phages such as T2, T4, and T6) targets the lipid A Kdo region deep in the LPS core, and a second variant targets the upper part(s) of the LPS core, which requires an intact inner LPS core for infectivity. The *Myoviridae* phage used in this study clusters with the latter group (Sørensen et al., 2020). We found genetic changes in the gene encoding glycosyltransferase required for the assembly of the LPS as well as in the genes encoding LPS inner core heptose (II) kinase (*waaY)* and heptosyltransferase I (*waaC*). Accordingly, as both *waaY* and *waaC* are essential for the LPS inner core, the nonsense mutations detected in these genes will most likely have an effect on the infectivity of an infecting phage. Either a direct effect as shown for phages utilizing the LPS as a receptor (Pagnout et al., 2019) or an indirect effect where *waa* mutation(s) interfere with the recognition of outer membrane protein phage receptors (Borin et al., 2021). At the same time, mutants with truncated LPS at the inner core have been shown to have attenuated *in vivo* virulence and to be more sensitive to antimicrobials (Hantke, 2020; Salazar et al., 2021).

The O-antigen biosynthesis operon has been shown to play a major role in *E. coli* phage resistance against *Myoviridae* phage T4 (Cowley et al., 2018) and *Demerecviridae* (previous *Siphoviridae*) phage T5 (Bertozzi Silva et al., 2016). In accordance with these previous observations, we found genetic changes (missense mutation, partial or complete gene loss) in four O-antigen operon genes encoding a glycosyltransferase, the O-antigen polymerase (*wzy*), a chain length determinant protein (*wzzB* gene) (Stenberg et al., 2005), and the O antigen flippase (*wzx* gene/*rbfX* gene), all of which could potentially confer phage resistance. These findings could support the LPS as a potential binding site for our phage.

In both Gram-negative and Gram-positive bacteria, the RNase adaptor protein RapZ plays a central role in regulatory pathway of glucosamine-6-phosphate (GlcN6P), an early and essential precursor in the synthesis of the bacterial cell envelope components, including peptidoglycan, LPS and colanic acid (Gonzalez et al., 2017). Recent studies have demonstrated that phage resistance in *E. coli* and *Staphylococcus aureus* can be acquired through mutation(s) in the *rapZ* gene, encoding RapZ (Azam et al., 2018; Mutalik et al., 2020; Zhou et al., 2021). Zhou et al. (2021) reported that mutation in the *rapZ* gene conferred *E. coli* phage resistance by inhibiting 93.5% phage adsorption. In this study, we similarly detected a missense mutation in the *rapZ* gene supporting its involvement in phage resistance against lytic *Myoviridae* coliphages. Moreover, in according with finding of Zhou et al. (2021), no *in vitro* fitness cost (measured by bacterial growth) was associated with the acquired resistance.

The polysaccharide capsule of pathogen *E. coli* K1 is an essential virulence factor and consist of polymers of sialic acid (NeuNAc). The *kps* gene cluster encodes six proteins, NeuDBACES, required for synthesis, activation, and polymerization of NeuNAc (Daines et al., 2000; Silver et al., 2001; Vimr et al., 2004). In this study, we detected partial gene loss of *neuD* (involved in the synthesis of sialic acid) (Daines et al., 2000), *neuA* (synthase involved in activation the sugar prior to polymerization) (Daines et al., 2000), and *neuE* (involved in synthesis and export of NeuAc) (Steenbergen and Vimr, 2008). The capsule is recognized as a receptor by some phages, such as K-specific coliphages and the *Myoviridae* coliphage phi92, which have virion-associated polysaccharide-degrading enzymes (Schwarzer et al., 2012; Latka et al., 2017). Scholl et al. (2005) showed that the expression of the *E. coli* K1 capsule physically blocks infection by phage T7, a phage that recognize LPS core as the primary receptor. Whether or not our *Myoviridae* phage can utilize the capsule as receptor needs to be investigated further. Nevertheless, as polysaccharide capsule is a key virulence factor, the interesting finding that ∼23% of the phage resistant isolates have lost part of one of the *neu* genes could add to the phage therapy potential of the infecting phage. Being as the infection could result in reduced virulence as well as competitiveness. Accordingly, (partial) loss of *neuE* may be associated with great fitness cost as up to ∼65% growth decrease was observed for the phage-resistant mutant strains. However, in all affected strains two or more other genetic changes were detected, strongly implying that further studies are needed to determine the exact effect of *neuE* loss alone and in combination with the other affected genes.

Even though we were able to connect some of the genetic changes in the mutant strains to known phage resistance mechanisms, most SNPs (*n* = 23) and gene losses (partial or complete) (*n* = 11) were found in a gene not previously linked to phage resistance. Among others, these gene encodes acetate kinase (essential for bacterial growth) (Schütze et al., 2020), Acyl-CoA dehydrogenase (involved in the beta-oxidation cycle of fatty acid degradation) (Campbell and Cronan, 2002), the MarR family transcriptional regulator (involved in numerous cellular processes, including stress responses, virulence, and efflux of harmful chemicals and antimicrobials) (Deochand and Grove, 2017), pyruvate kinases (essential for the regulation of the glycolytic pathway) (Valentini et al., 2000), a tetratricopeptide repeat (TPR) protein (involved in various biological processes and mediates protein-protein interactions) (Cerveny et al., 2013), uridylyltransferase (involved in nitrogen regulation) (Zhang et al., 2010) as well as several hypothetical proteins. We found loss of the gene or mutation in an acetate kinase, pyruvate kinase, TPR protein and uridylyltransferase as the sole genetic change indicating that the phage-host interaction might be more complex that previous thought. Interestingly, partial or complete loss of one of two genes (*group_67* and *group_237*) encoding hypothetical proteins was detected in a great number of phage-resistant mutant strains, and as sole resistance mechanisms in some. Loss of *group_67* gene as sole resistance mechanism resulted in an average fitness cost (growth reduction) of only 6.3%. Similarly, loss of the *neuE* gene as sole resistance mechanism resulted in an average fitness cost of only ∼3.9%. However, the greatest fitness cost was observed for the mutant strain that had lost both the *group_67* and *neuE* (65.2%) or both genes in combination with a point mutation in the *phnD* gene (57.0%), indicating that a combination loss of *group_67* and *neuE* might have an additive effect on the fitness cost. The point mutation in *phnD* was only observed in one mutant and only in combination with *group_67* and *neuE* gene loss. Only one mutant had lost the *group_237* gene as sole resistance mechanisms and suffered a great fitness cost of 59.1%. Moreover, an average fitness cost of 23.2% was observed for the 10 mutant strains with *group_237* gene loss, suggesting that while mutation in this gene might confer phage resistance, the resistance comes with a cost for the host bacterium. Furthermore, one mutant had lost genes encoding both *group_237* and *group_67* and suffered a fitness cost (34.6%), supporting the essential role of *group_237* and the potential additive effect of *group_67* gene loss. However, as additional genetic changes (potentially related to phage resistance) were detected in most of both the *group_67* and *group_237* mutant strains. We tried to decipher the potential function of the hypothetical proteins, using PANDA (Wang et al., 2018) and LocTree3 (Goldberg et al., 2014), however, we could not find any motifs that could give an indication (data not shown). Also, the role of these proteins in *E. coli* phage resistance needs to be further investigated.

A nonsense mutation was detected in the gene encoding the YbjT protein. This protein has been shown to be physically tethered to the inner membrane of *E. coli* and part of the metabolic pathway involved in the biogenesis of the bacterial cell envelope (Hu et al., 2009). However, as this genetic change was not the only one detected in the affected strain, its potential involvement in phage resistance remains to be investigated. Finally, six different synonymous SNPs were identified in this study. Although unlikely, these mutations may still play a role in phage resistance as synonymous mutations can affect cellular processes such as translation efficiency or mRNA structures, depending on the gene affected (Plotkin and Kudla, 2011).

CRISPR-Cas systems are found among ∼36% of bacteria and confer a sequence specific adaptive immunity against invading foreign DNA, including phages (Pourcel et al., 2020). Previous studies have reported varying findings when it comes to phage resistance conferred by acquired CRISPR spacer(s). As opposed to findings of Denes et al. (2015) where no CRISPR immunity was observed in any of the spontaneous phage-resistant *Listeria* mutant strains, in most of the phage-resistant *Streptococcus* mutant strains one or two CRISPR spacer(s) were acquired (Levin et al., 2013). In this study, we found three phage-resistant strains with a newly acquired CRISPR spacer sequence that matched the invading phage genome. This spacer was found in a short CRISPR array, only consisting of this one spacer (evidence level 1), which makes it difficult to determine if this array is a false CRISPR-like element or a true CRISPR. However, the lack of similar repeats in larger CRISPR arrays, associated *cas* genes and leader sequence upstream of the CRISPR array, are indications that the detected CRISPR spacer in the three phage-resistant mutant strains most likely is a false positive (Couvin et al., 2018). Moreover, two out of the three mutant strains had acquired one or three genetic changes in addition to the CRISPR-like spacer acquisition, including partial loss of the *group_237* gene or partial loss of *neuE*, complete loss of the *group_67* gene and a silent point mutation in a hypothetical protein. As discussed earlier, the partial and/or complete gene loss(es) are more likely to explain the resistance observed.

Phages have shown to be able to evolve to counteract bacterial antiviral mechanisms, such as inhibition of phage adsorption, R-M systems, CRISPR-Cas systems and phage escape strategies (Samson et al., 2013; Koskella and Brockhurst, 2014). Such adaptation can be conferred by point mutations in specific genes, such as receptor binding proteins (RBPs) and/or tail fibers, genome rearrangement, and genetic exchange with other viral or bacterial genomes to acquire new traits (Samson et al., 2013). Phage genes involved in host recognition are among the fastest evolving phage genes due to the selection pressures conferred by the phage-bacterium co-evolution (Samson et al., 2013; Borin et al., 2021). Meyer et al. (2012) showed that a lytic coliphage was able to evolve as such that it could use an alternative receptor after 8 days of co-culture with a resistant bacterial host. Similarly, Wandro et al. (2019) showed that after 8 days of co-culture the lytic *Enterococcus* Phage EfV12-phi1was able to combat phage-resistance through adaptation of the tail fiber. Hall et al. (2011) were able to detect adaptation in *Pseudomonas* phage SBW25Φ2 tail fiber protein and structural protein after only 2 and 4 days of co-culture, respectively. As opposed to these findings, in this study we did not detect any genetic changes in phages co-cultured with phage-resistant strains. However, this is most likely a reflection of a too short co-culture incubation period (<24 h) rather than the ability of the phage to co-evolve to bypass the phage resistance.

Understanding the phage-host interactions provides insight into the phage-host interaction and dynamics and may lead to new strategies for the development and application of successful phage therapy (Chaturongakul and Ounjai, 2014; Federici et al., 2021). Furthermore, the understanding of the interactions makes it possible adapt to phage selection toward the desired outcome (Stone et al., 2019). This includes selecting optimal phage(s) that can overcome host phage-resistance mechanisms, select for attenuated virulence, for impaired fitness/growth, and/or select for increased susceptibility to antimicrobials. Further studies comparing how different phages select for resistant bacteria may also lead to better understanding on how bacteria react on phage infection. Although the full complexity of the interactions cannot be captured, *in vitro* experiments can still provide essential information needed for further application in a therapeutic setting (*in vivo/in situ*) (Casey et al., 2018).

For 44 phage-resistant strains no detected genomic changes differentiated them from the WT strain. This could be caused by both laboratory issues, such as non-resistant strains were erroneously defined as true resistant mutants based on AUC values, or actual variations that were missed due to genetic variation within discarded repetitive regions identified by NUCmer or partly loss off genes of which the consequence on the overall gene function were not investigated.

Our experiments were conducted *in vitro* and thus caution should be used when interpreting our findings for *in vivo* applications. The co-evolutionary interactions, including phage resistance, observed in laboratory experiments can differ from the highly complex interactions found in natural environments, which may influence the ecology and evolution of both phages and their hosts (Laanto et al., 2017).

## Conclusion

In conclusion, under selective pressure of virulent phages, bacterial strains of *E. coli* can acquire one or more spontaneous mutations or gene losses that confer phage resistance *in vitro*. The majority of detected phage-resistant mutant strains from this study were shown to resist phage infection through mechanisms related to phage adsorption inhibition. Interestingly, we also found several new genes, including two encoding hypothetical proteins, that could potentially play a role in *E. coli* phage resistance. There were no indications that the infecting phages were able to overcome the phage resistance. Nevertheless, as the initial infection targeted known *E. coli* virulence factors, such as OMPs and the LPS, and thus, potentially decreased the APEC virulence, the infecting phage still possessed desirable traits for phage therapy application. Furthermore, in many cases phage resistance was associated with fitness cost for the affected mutant strain resulting in up to ∼65% decrease in growth. Thus, this study provides valuable information about the interactions between virulent coliphages and their host, which may aid prediction of the phage-host interaction outcome and future development of a successful phage therapy.

## Data Availability Statement

The datasets presented in this study can be found in online repositories. The names of the repository/repositories and accession number(s) can be found below: https://www.ncbi.nlm.nih.gov/, PRJNA745212.

## Author Contributions

PS carried out the experimental work and wrote the article. PS and SB performed the genome sequence analysis with support from MS. MS provided sequencing facilities. AG and HI co-supervised the work. PB supervised the research. All authors contributed to manuscript editing and have approved of the article before submission.

## Conflict of Interest

The authors declare that the research was conducted in the absence of any commercial or financial relationships that could be construed as a potential conflict of interest.

## Publisher’s Note

All claims expressed in this article are solely those of the authors and do not necessarily represent those of their affiliated organizations, or those of the publisher, the editors and the reviewers. Any product that may be evaluated in this article, or claim that may be made by its manufacturer, is not guaranteed or endorsed by the publisher.

## Footnotes

1
https://github.com/ssi-dk/bifrost

2
https://github.com/tseemann/abricate

3
https://crisprcas.i2bc.paris-saclay.fr/CrisprCasFinder/Index
