# Supplementary material for: Spontaneous Phage Resistance in Avian Pathogenic Escherichia coli
Source: Front Microbiol. 2021 Dec 13;12:782757. doi: 10.3389/fmicb.2021.782757 (PMC8711792; doi:10.3389/fmicb.2021.782757)
Supplement: Supplementary file 1 [file Data_Sheet_1.zip › Supplementary Figure S1.DOCX]

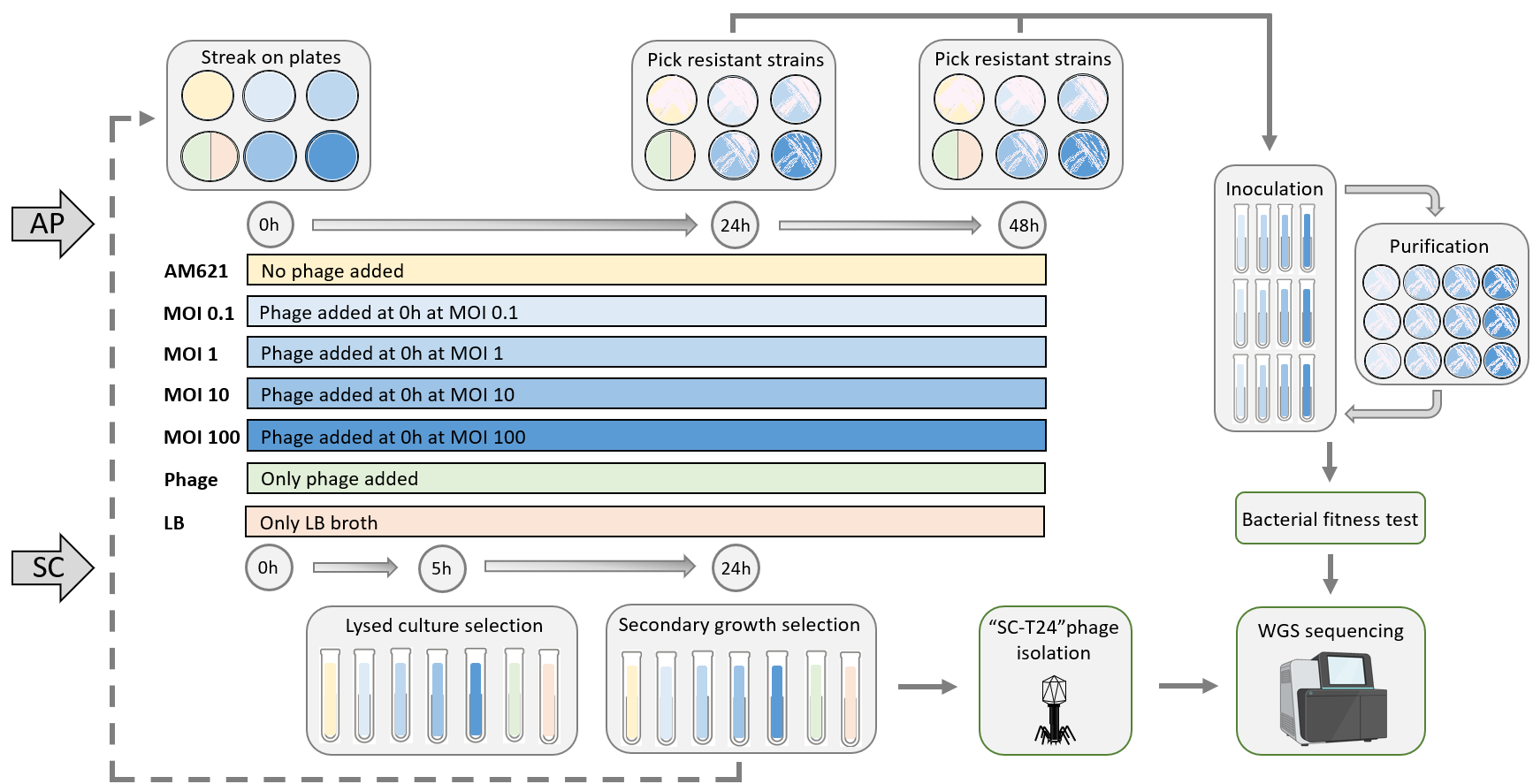


**Supplementary Figure 1.** Experimental setup of the isolation of phage resistant APEC strains by secondary culture (SC) or agar plate (AP) methods. At time 0 h, susceptible AM621 WT cultures were infected with phage at four different multiplicities of infection (MOIs): MOI 0.1, MOI 1, MOI 10, and MOI 100. An un-infected control (AM621) and negative controls (Phage and LB) were included. AP approach: suspensions were streaked directly onto LB agar plates and incubated for 48 h. Resistant colonies were picked after 24 h and 48 h of incubation, grown in LB broth and purified by three consecutive streakings on LB agar plates. SC approach: Remaining “0h” suspensions were incubated for 5 h. Suspensions exhibiting lysis were incubated for additional 24 h. If subsequent (secondary) growth was observed, suspensions were streaked onto plates and processed as described for the AP approach. Phage resistant strains were named according to experimental setup (AP or SC and MOI. Experimental steps following the isolation are outlined in green.
