# Supplementary material for: Spontaneous Phage Resistance in Avian Pathogenic Escherichia coli
Source: Front Microbiol. 2021 Dec 13;12:782757. doi: 10.3389/fmicb.2021.782757 (PMC8711792; doi:10.3389/fmicb.2021.782757)
Supplement: Supplementary file 1 [file Data_Sheet_1.zip › Supplementary Figure S2.DOCX]

**(B)**

**(A)**


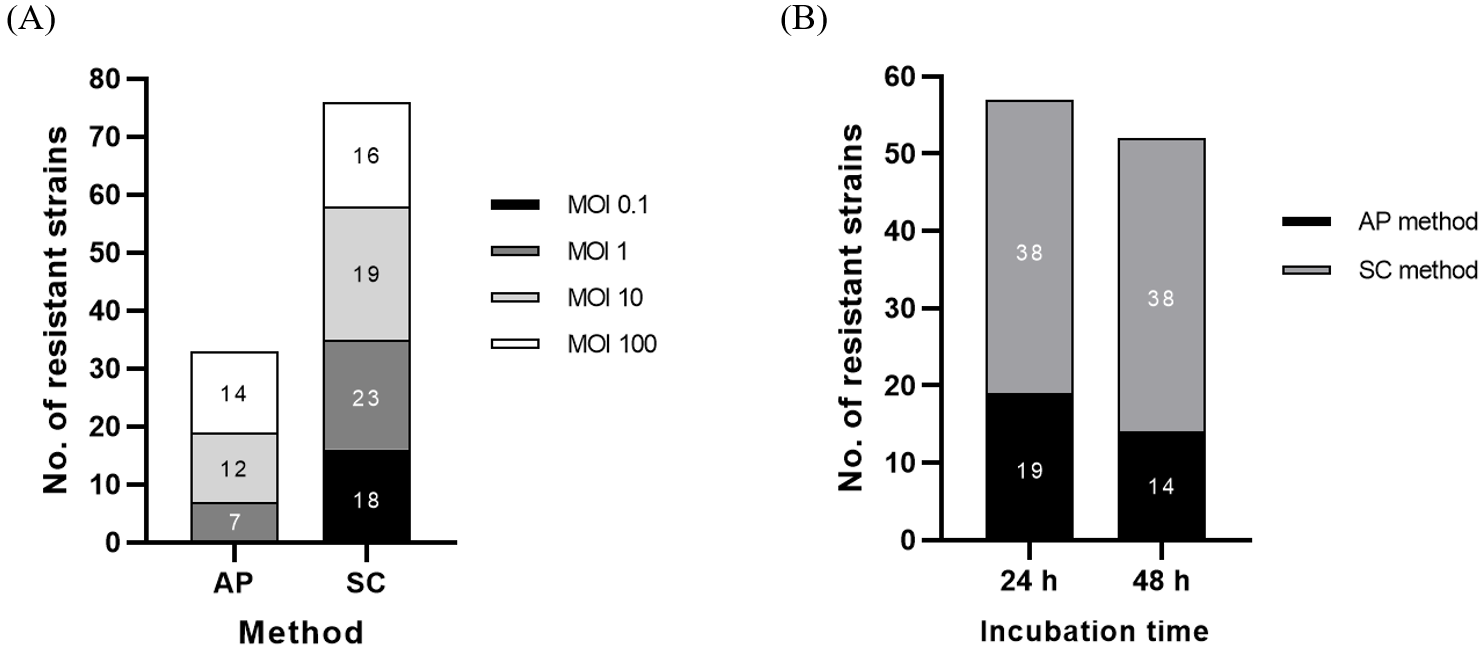


**Supplementary Figure 2.** Phage-resistant strains isolated from pathogenic *E. coli*  by secondary culture (SC) or agar plate (AP) methods**. (A)** Number of resistant strains for each method and multiplicity of infection (MOI) using the AP or SC method, including MOI 0.1 (black), MOI 1 (dark grey), MOI 10 (light grey), and MOI 100 (white). **(B)** Number of resistant strains isolated for each timepoint (24 h or 48 h of incubation) for the AP method (black) and SC method (grey). The number of resistant strains is indicated for each combination. The numbers were obtained based on six repeated isolation experiments.
