# Supplementary material for: Spontaneous Phage Resistance in Avian Pathogenic Escherichia coli
Source: Front Microbiol. 2021 Dec 13;12:782757. doi: 10.3389/fmicb.2021.782757 (PMC8711792; doi:10.3389/fmicb.2021.782757)
Supplement: Supplementary file 1 [file Data_Sheet_1.zip › Supplementary Table S3.DOCX]

**Supplementary Table S3** | Overview of evidence level 1-CRISPR spacers detected in phage-resistant strains.

| **Spacer #** | **Length (bp)** | **Spacer sequence** | **BLAST hit** | **GenBank** | **Annotation** |
| --- | --- | --- | --- | --- | --- |
| 1 | 40 | GCGCTGCGGGTCATTTTTGAAATTACCCCCGCTGTGCTGT | *Escherichia coli* strain SB0258h1 | CP071954.1 | General stress protein |
| 2 | 54 | GCCGTTGCCGAATGTAGGCCGGATAAGGCGTTCACGCCGCATCCGGCAACCAGC | *Escherichia coli* strain EcPF5 | CP054236.1 | - |
| 3 | 34 | CTGTAATTTTCATGAAAGGTGGATGGCTGCGCAC | *Escherichia coli* strain CP8-3_Sichuan plasmid pCP8-3-IncX1 | CP053740.1 | Plasmid (pCP8-3-IncX1) |
| 4 | 38 | CGGACGCAGGATGGTGCGTTCAATTGGACTCGAACCAA | *Escherichia coli* strain LWY6 | CP072204.1 | tRNA-Val |
| 5 | 58 | GGAGCCAGAAGAACAGATTGATCCGCGCAAAGCCGCCGTCGAAGCTGCTATTGCCCGT | *Escherichia coli* strain SCU-487 | CP054454.1 | Electron transport complex subunit RsxC |
| 6 | 53 | TTTCAAGTATTGTAAAACATTTGATGCAATCGCTTATATTGCCGAATCTTTTG | *Escherichia* phage vB_EcoM_G29 | MK327940.1 | PinA peptidase inhibitor |
| 7 | 24 | GGGGGGGGGGGGGGGGGGGGTTTG | - | - | - |
| 8 | 27 | CCCCCCCCCCCCCCCCCCCCCCCCCCC | - | - | - |
